# FKBP39 Controls the Larval Stage JH Activity and Development in *Drosophila melanogaster*

**DOI:** 10.3390/insects13040330

**Published:** 2022-03-28

**Authors:** Xinyu Wang, Ying Zhou, Jianwen Guan, Yang Cheng, Yingying Lu, Youheng Wei

**Affiliations:** 1 Joint International Research Laboratory of Agriculture and Agri-Product Safety, the Ministry of Education of China, Yangzhou University, Yangzhou 225009, China; w957017461@163.com (X.W.); zy1148174033@163.com (Y.Z.); gjw2547682446@163.com (J.G.); chengy@yzu.edu.cn (Y.C.); lyying@yzu.edu.cn (Y.L.); 2 College of Bioscience and Biotechnology, Yangzhou University, Yangzhou 225009, China

**Keywords:** FK506-binding protein 39kD (FKBP39), juvenile hormones pathway, Drosophila, qRT-PCR, rp49

## Abstract

**Simple Summary:**

Two endocrine hormones, ecdysone and juvenile hormone (JH), control insect development and reproduction. Some studies in the literature have suggested that FKBP39 functions as a transcriptional factor and regulates the JH pathway in Drosophila. However, the physiological roles of FKBP39 are still elusive. To determine the FKBP39 roles in vivo, we first developed an antibody to check the FKBP39 expression pattern and then detected JH activity-related phenotypes in *fkbp39* mutants, such as pupariation, reproduction, and Kr-h1 expression. We found that FKBP39 expresses at a high level and controls JH activity at the larval stage. Moreover, we found that rp49, the most widely used reference gene for Real-time quantitative PCR (qRT-PCR), significantly decreased in the *fkbp39* mutant. This work will provide valuable information for studies on JH activity and insect development.

**Abstract:**

FK506-binding protein 39kD (FKBP39) localizes in the nucleus and contains multiple functional domains. Structural analysis suggests that FKBP39 might function as a transcriptional factor and control juvenile hormone (JH) activity. Here, we show that FKBP39 expresses at a high level and localizes in the nucleolus of fat body cells during the first two larval stages and early third larval stage. The *fkbp39* mutant displays delayed larval-pupal transition and an increased expression of *Kr-h1*, the main mediator of the JH pathway, at the early third larval stage. Moreover, the *fkbp39* mutant has a fertility defect that is independent of JH activity. Interestingly, the expression of *rp49*, the most widely used reference gene for qRT-PCR in Drosophila, significantly decreased in the *fkbp39* mutant, suggesting that FKBP39 might regulate ribosome assembly. Taken together, our data demonstrate the expression pattern and physiological roles of FKBP39 in Drosophila.

## 1. Introduction

**Figure 1:**
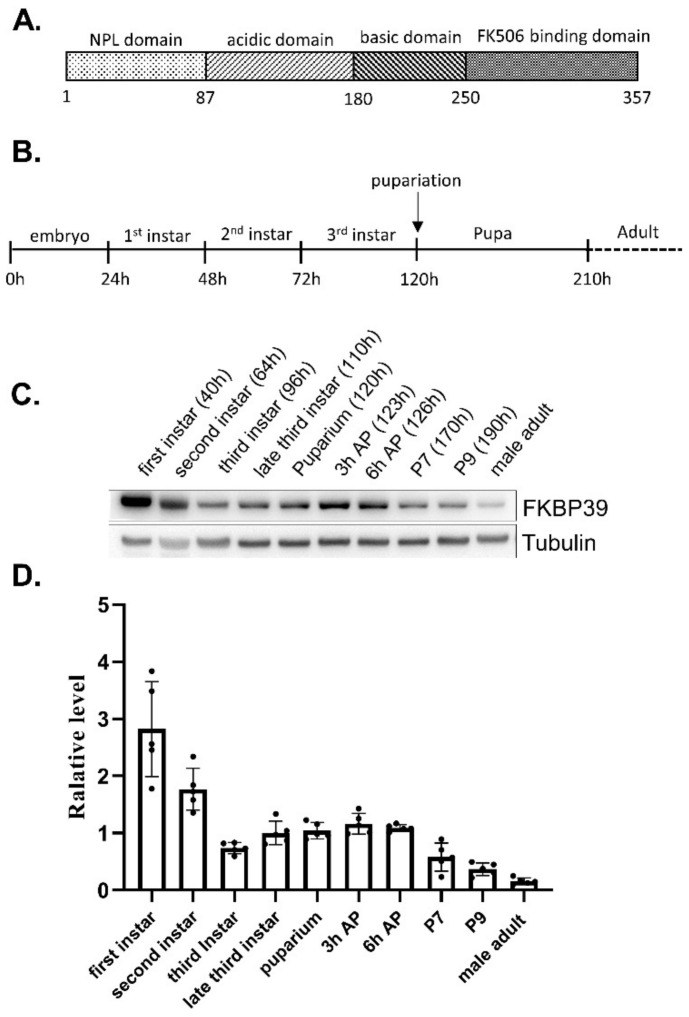
FKBP39 expression during Drosophila development. (**A**) The schematic representation of the putative functional regions of FKBP39. (**B**) The scheme of the fly development stages. (**C**) Western blot analysis of the FKBP39 protein at different development stages. AP, after puparium; P, pupal stage. The pupal stages are according to stages of metamorphosis in Drosophila [22]. The time points of the sample collection are shown. α-Tubulin was used as a loading control for the Western blot. (**D**) Quantification of FKBP39 levels relative to α-Tubulin. Error bars represent the S.D. of five independent experiments.

FK506-binding proteins (FKBPs), a large group of conserved proteins, contain the peptidyl-prolyl cis-trans isomerase (PPIase) motif in the FKBP domain, which binds the immunosuppression drug FK506 [1,2]. Besides the FKBP domain, most FKBPs have additional domains, which make the FKBP members display various functions such as transcriptional regulation and histone modification [3,4]. FKBP39, also named FK506-bp1, is the first identified FKBP in Drosophila [5]. The Drosophila FKBP39 consists of a nucleoplasmin-like (NPL) domain in the N terminal region, an FKBP domain in the C terminal region, and a highly flexible linker domain (Figure 1A) [3,6]. The NPL domain of FKBP39 itself forms a pentameric structure implicating its role in chromatin regulation [7]. However, Kozlowska et al. showed that the NPL domain of the full-length FKBP39 formed a tetramer, linked by a disordered acidic and basic fragment and an FKBP domain [8]. The multiple domains, especially the highly disordered linker between FKBP and NPL, make the structure of FKBP39 very flexible and dynamic, which may provide the foundation of its multiple functions. In addition to forming a tetramer, the full-length FKBP39 molecules existed as monomers and dimers, suggesting that FKBP39 forms multiple complexes and performs different functions [8]. FKBP39 plays an essential role in developmental autophagy and cell metabolism. Li et al. have shown that the two nuclear proteins, FKBP39 together with 21 kDa calponin-like protein (Chd64), form a multi-protein complex that is dynamically associated with the juvenile hormone response element (JHRE), suggesting that FKBP39 might regulate ecdysone and JH pathways in Drosophila [9]. Juhasz et al. found that FKBP39 inhibits developmental autophagy by regulating the transcription factor Forkhead O (FOXO) pathway in the Drosophila larval fat body [10].

As a complete metamorphosis insect, Drosophila developmental transition is tightly regulated by levels of the two primary endocrine hormones ecdysone and juvenile hormone (JH), which have opposite functions [11,12]. The ecdysone works through its receptor ecdysone receptor (EcR) and ultraspiracle (USP), which binds to the ecdysone response elements (EcRE) located in the promoter regions of target genes such as the transcription factors *Broad* and *Eip93F* (*E93*), and promotes metamorphosis, whereas the JH together with its receptor Methoprene-tolerant (Met) and Germ cell-expressed (Gce) binds to JH response elements (JHRE) located in the promoter regions of target genes such as *Kr-h1* and prevents metamorphosis [13,14]. JH also prevents metamorphosis by repressing ecdysone biosynthesis and signaling [15]. The depletion of JH activity in Drosophila results in pupal lethality, meaning that JH is not essential for larval development but indispensable for metamorphosis [16]. Moreover, increased JH activity delayed the onset of pupariation [14,16]. The Krüppel homolog 1 (Kr-h1), a zinc-finger transcription factor, is the main effector of JH action and plays an essential role in insect metamorphosis [17,18]. In addition to regulating JH activity directly, Kr-h1 suppresses the expression of ecdysone target genes. Thus, Kr-h1 mediates the crosstalk between JH and ecdysone pathways [15,19]. The expression level of Kr-h1 is high at the early stage of each larval instar for larval maintenance. Consistent with the essential roles of JH activity in metamorphosis, *Kr-h1* mutant flies died at pupation with delayed pupariation. The inhibition of Kr-h1 activity accelerates the beginning of pupariation, whereas overexpression Kr-h1 blocks development at the larval stage [11,15,16]. These results suggest that the function of Kr-h1 in larval development is complicated.

Here, we demonstrate the expression pattern and physiological roles of FKBP39 in Drosophila. The FKBP39 expresses at a high level and localizes in the nucleus at the early larval stage. The *fkbp39* mutant displays a delayed larval-pupal transition and decreased fertility. Interestingly, the expression of *rp49*, the most widely used reference gene for qRT-PCR analysis, decreased in the *fkbp39* mutant. Moreover, the expression of *Kr-h1* increased in the *fkbp39* mutant. Our work suggests that FKBP39 controls JH activity and plays an essential role in Drosophila development.

## 2. Materials and Methods

### 2.1. Drosophila Strains

The stock CG-Gal4 (BDSC#7011) was obtained from Bloomington Stock Center. The stock *fkbp39^1^* was described previously, and *y*,*w* was used as a control [20]. All fly stocks were maintained on BDSC standard cornmeal medium at 25 °C, 60% humidity.

### 2.2. Generation of the Anti-FKBP39 Antibody

The nucleotide sequence of the FKBP39 fragment (amino acid sequence from 166 to 357, NP_524364) was amplified from Drosophila cDNA using the following primers. Fkbp39 forward: ACAGCAAATGGGTCGCGGATCCATGTCGATGTTTTGGGGT; Fkbp39 reverse: CTTGTCGACGGAGCTCGAATTCCTAATGCACAGCTTTCAGT. The fragment was inserted into PET-28a to generate the plasmid for expression in E. coli. The fragment of FKBP39 tagged with 6*His was purified with Ni-NTA (CWbiotech). The mouse polyclonal antibody was generated using a purified FKBP fragment (Beijing Protein Innovation). 

### 2.3. Western Blot Analysis

The eggs laid by *y*,*w* flies were collected and cultured on standard food to different developmental stages, and they were then homogenized in an RIPA buffer containing complete protease inhibitors and phosphatase inhibitors (Roche). For the Western blot, antibodies were used at the following concentrations: mouse anti-α-tubulin at 1:10,000 (Beyotime, #AF0001); mouse anti-FKBP39 at 1:2000 (generated in this work). Quantitative measurements of Western blots were performed using ImageJ software.

### 2.4. Developmental Time Analysis, Viability Analysis, and Fertility Analysis

The eggs laid by *y*,*w* or *fkbp39^1^/TM6,Tb* heterozygous flies were collected within 2 h and cultured on standard food to the puparium. The homozygous *fkbp^1^* larvae were selected with the absence of the *Tubby* phenotype, and the developmental time from egg to puparium was recorded for each fly. The newly formed puparia of *y*,*w* or *fkbp39^1^* were collected, and the developmental time from puparium to adult was recorded for each fly. 

Five pairs of *fkbp39^1^*/*MKRS*,*Sb* were crossed, and the heterozygous and homozygous flies in the next generation were counted to calculate the ratio and judge the viability of *fkbp39^1^*. In these crosses, the MKRS (third chromosome balancer) was identified by the Sb marker.

Five pairs of flies were cultured under standard conditions for three days and then transferred to the culture tube. The number of eggs was counted each day. The number of hatching eggs to the first instar larvae was also checked and counted from the second to the fifth day after egg-laying.

### 2.5. RNA Isolation and mRNA Analysis

RNA isolation and qRT-PCR were performed as previously described [21]. The following primers were used for qPCR. Actin forward: GCGTCGGTCAATTCAATCTT; Actin reverse: AAGCTGCAACCTCTTCGTCA; Tub 84b forward: CGTTGGTGAGGGTATGGAGG; Tub 84b reverse: TGATTTCGACGGTTACCCCG; Rps20 forward: GTTCGCTGGAGAATGTGTGC; Rps20 reverse: CAGGTCTTGGAACCCTCACC; Kr-h1 forward: ATCCGCTCTACCCAATCCG; Kr-h1 reverse: AGCCTTCTCCGAATCCACCT; rp49 forward: GCCGCTTCAAGGGACAGT; rp49 reverse: CGATCTCGCCGCAGTAAA.

### 2.6. Immunofluorescence 

Immunofluorescence staining was performed using a mouse anti-FKBP39 (1:500) antibody. Anti-mouse Alexa Fluor 594 secondary antibodies (Invitrogen) were used at a dilution of 1:1000. Nuclei were visualized by staining the DNA with DAPI (Invitrogen). Images were acquired using a Zeiss 880 confocal microscope.

### 2.7. Statistical Analyses

Data are reported as the mean ± SD of at least three independent experiments and analyzed using a two-tailed *t*-test.

## 3. Results

### 3.1. The Developmental Pattern of the FKBP39 Protein

To determine the FKBP39 expression pattern in Drosophila, we developed an antibody of FKBP39 for immunostaining and immunoblot (Supplemental Figures S1 and S2). We collected the samples at different developmental stages and detected the FKBP39 expression (Figure 1B–D). We found that the expression level of FKBP39 at the first larval stage was highest, followed by a decrease in the second and early third instar, after which it remained at an intermediate level through early metamorphosis (the prepupa) and then declined to a very low level in the male adult (Figure 1C,D). This result suggests that the FKBP39 expression level is stage-specific during Drosophila development.

**Figure 2:**
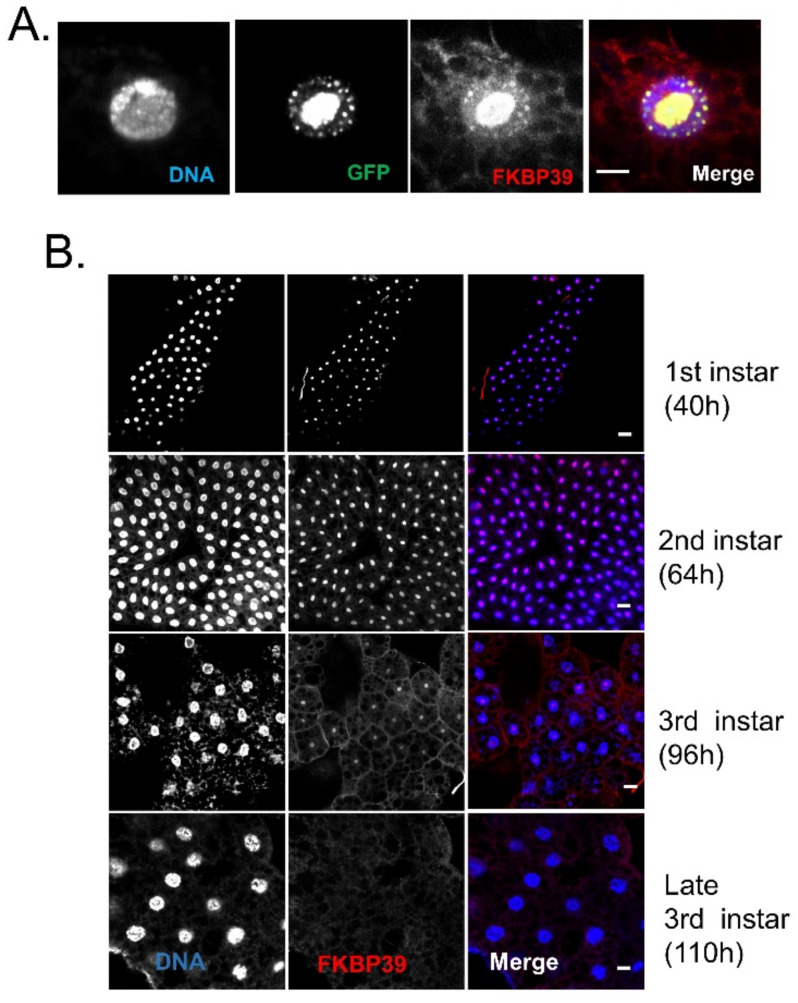
The nuclear localization pattern of FKBP39 in the fat body. (**A**) The fat body from CG-GAL4; UAS-GFP-FKBP39 were dissected and stained with FKBP39 antibody (red). (**B**) Different stages of fat bodies were stained with FKBP39 antibody (red) and DAPI (blue). Bar: 10 μm.

Next, we would like to examine the FKBP39 localization in Drosophila cells. Li et al. had identified the endogenous FKBP39 in isolated Drosophila cell nuclear proteins by LC-MS/MS analysis [9]. Another two studies reported that the exogenous fluorescent protein-tagged FKBP39 was localized in the nucleus [7,10]. More recently, Kozlowska et al. showed that the exogenous YFP-FKBP39 was localized in the nucleolus in human COS-7 cells [8]. Here, we used the fat body to determine the FKBP39 localization in vivo. The Drosophila fat body, analog to the vertebrate adipose tissue and liver, is a single-layer tissue that is widely used to determine protein localization. The fat body is an excellent model for tissue remodeling during metamorphosis controlled by endocrine hormones, JH, and ecdysone [23]. Thus, we overexpressed GFP-tagged FKBP39 in the fat body and found that the GFP-FKBP39 was localized in part of the nucleus (Figure 2A). The FKBP39 antibody stains the same location as GFP-FKBP39, suggesting that the FKBP39 antibody can recognize FKBP39 protein in vivo. Using the FKBP39 antibody, we checked the endogenous FKBP39 expression at different developmental stages (Figure 2B). Similar to the GFP-FKBP39, the endogenous FKBP39 was mainly localized in part of the nucleus at the second and early third instar. Interestingly, the nuclear localization of FKBP39 significantly declined to an undetectable level at the late third instar. The stage-specific expression level and nuclear localization might be associated with its function during development. 

### 3.2. FKBP39 Plays an Essential Role in Drosophila Development

To determine the physiological roles of FKBP39 in Drosophila development, we detected the viability and fertility of the *fkbp39* mutant, *fkbp39^1^* [20]. We collected the eggs laid from heterozygous (*fkbp39^1^/MKRS*) flies and cultured them on standard food to check the viability of the *fkbp39* mutant. The ratio of eclosed *fkbp39^1^* homozygous adults to heterozygous adults was close to the expected Mendelian ratio, which suggested that the *fkbp39^1^* was fully viable (Table 1).

**Figure 3:**
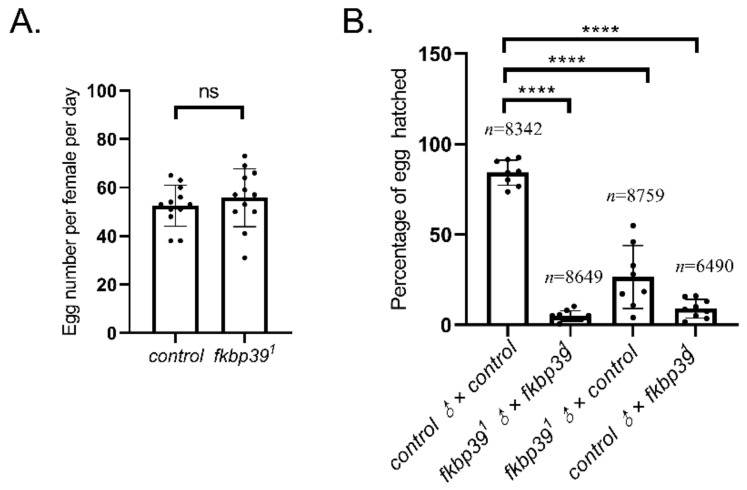
The *fkbp39* mutant flies have fertility defects. (**A**) The number of eggs laid by wild-type or *fkbp39^1^* females. Error bars represent the S.D. of independent experiments. (**B**) The percentage of eggs successfully eclosed from the indicated fly genotype crossing. Error bars represent the S.D. of eight independent experiments. n is the total number of eggs examined. **** *p* < 0.0001; ns, not significant.

To trace the fertility of the *fkbp39* mutant, the number of eggs laid and the ratio of egg-hatching were checked. The egg-laying of *fkbp39^1^* was comparable to the wild type (Figure 3A). However, most of the eggs laid by *fkbp39^1^* homozygous flies were unhatched, suggesting that the *fkbp39^1^* was semi-sterile. We crossed both female and male *fkbp39^1^* with wild-type flies to detect whether the sterility was due to female or male defects. The fertility decreased in both female and male *fkbp39^1^* (Figure 3B). This result showed that the gametes produced by the *fkbp39* mutant were defective. 

**Figure 4:**
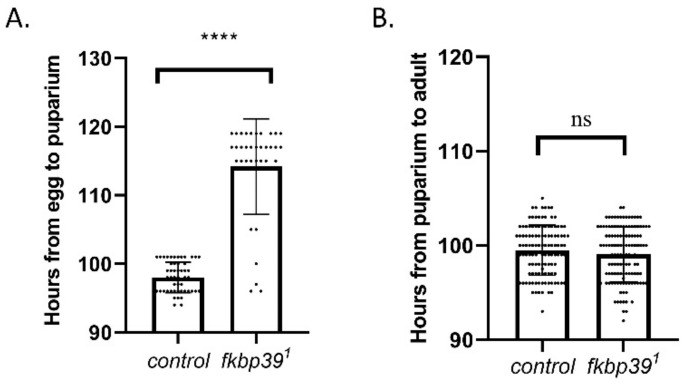
FKBP39 controls Drosophila development. (**A**) The developmental time from egg to puparium in wild type or *fkbp39^1^*. (**B**) The developmental time from puparium to adult in wild type or *fkbp39^1^*. Error bars represent the S.D. of indicated values. **** *p* < 0.0001; ns, not significant.

Although most of the homozygous fkbp39 were eclosed (Table 1), we found that the homozygous *fkbp39^1^* flies eclosed later than the heterozygous flies, suggesting a development delay in the *fkbp39* mutant. To determine which developmental stage in the *fkbp39* mutant was affected, eggs from wild-type or heterozygous specimens (*fkbp39^1^*/*MKRS, Sb*) were collected within 2 h and allowed to develop under the same environment (25 °C, 60% humidity). The longevities of the wild-type and homozygous *fkbp39^1^* flies at the larval and pupal stages were quantified. The time from egg to pupariation was longer in the *fkbp39* mutant compared to the control, whereas the time from pupariation to adult did not change (Figure 4A,B). These results suggest that FKBP39 promotes larval development in Drosophila.

### 3.3. FKBP39 Controls rp49 Expression

**Figure 5:**
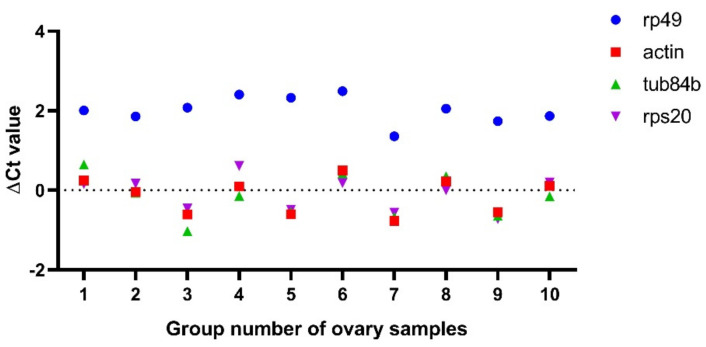
*Rp49* decreased expression in *fkbp39* mutant ovaries. The ∆Ct values of the indicated genes between wild type and *fkbp39^1^* were detected by qRT-PCR.

Next, we wanted to detect whether some gene expressions changed in the *fkbp39* mutant using the real-time quantitative reverse transcription PCR (qRT-PCR) method. Ribosomal protein L32 (*RPL32)*, also named *rp49*, is the most widely used reference gene for qRT-PCR in Drosophila. Surprisingly, we found that the Ct value of *rp49* always increased in the *fkbp39* mutant ovaries compared to the control, when we used an equal amount of total RNA for the qRT-PCR. We speculated that the loss of FKBP39 might affect *rp49* expression. To test this hypothesis, we detected the expression of three housekeeping genes, *Actin*, *Tub84b,* and *RPS20* [24]. We compared the ∆Ct value of these genes’ qRT-PCR amplification between the control and *fkbp39^1^* in 10 groups of the Drosophila ovary samples, using the same amount of RNA for each group. The ∆Ct values of *Actin*, *Tub84b,* and *Ribosomal protein S20* (*RPS20*) between the control and *fkbp39^1^* were almost identical and close to 0. However, the ∆Ct values of *rp49* were always higher than 0, and the average value was about 2 (Figure 5), which meant that the expression of *rp49* decreased 4-fold in *fkbp39^1^*. We also detected *rp49* expression in larvae and pupae and obtained similar results, suggesting that *rp49* was not suitable for normalization in the FKBP39 analysis.

### 3.4. FKBP39 Controls the Expression of Kr-h1 Specifically at the Larval Stage

**Figure 6:**
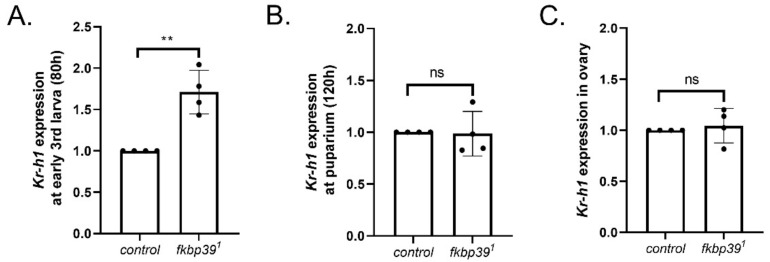
FKBP39 controls *Kr-h1* expression. (**A**) Fold change of the *kr-h1* mRNA at early 3rd instar larval stage in *fkbp39* mutant. (**B**) Fold change of the *kr-h1* mRNA at the puparium in *fkbp39* mutant. (**C**) Fold change of the *kr-h1* mRNA in *fkbp39* mutant ovary. *Actin* is used for normalization. Error bars represent the SD of four independent experiments. ** *p* < 0.01; ns, not significant.

The occurrence of larval molting and metamorphosis is accurately controlled by JH and ecdysone activity [11]. Previous studies have suggested that FKBP39 might control JH activity [9]. To determine the effect of FKBP39 on JH activity in vivo, we detected the expression of *Kr-h1*, the main mediator of the JH pathway, in the *fkbp39* mutant using *Actin* as a reference gene. The *Kr-h1* expression significantly increased at the early third instar and did not change at pupariation in the *fkbp39* mutant, which is consistent with the phenotype that increased the longevity of the larval stage and with the unchanged longevity of the pupal stage in the *fkbp39* mutant (Figure 6A,B). To determine whether the sterility of the *fkbp39* mutant was associated with the JH pathway, we checked the *Kr-h1* expression in the ovary and found that the *Kr-h1* expression in the *fkbp39* mutant did not significantly change (Figure 6C). These results suggest that FKBP39 might regulate JH activity specifically at the larval stage.

## 4. Discussion

Similar to other members of the FKBP family, FKBP39 contains multiple functional motifs, including the NPL domain and a highly disordered linker [6,8]. Here, we report that FKBP39 accelerates the larval-pupal transition and is required for fertility. In addition, we show that FKBP39 regulates the expression of *rp49* and *Kr-h1*.

Western blots showed that FKBP39 protein is high in the first two larval instars, then declines to intermediate levels during the final larval instar and prepupal period and to very low levels in late adult development. This pattern is quite similar to the developmental proteome data for FKBP39 and is to be expected from the ModEncode data for *fkbp39* mRNA seen in FlyBase (www.flybase.org, accessed on 16 March 2022). A previous study showed that overexpression of FKBP39 inhibited developmental autophagy in Drosophila fat body cells at the late third larval stage [10]. Developmental autophagy is essential for fat body degradation during metamorphosis. The observed decreased FKBP39 at the late third larval stage might help to promote developmental autophagy initiation in the fat body.

Our finding that *Kr-h1* expression is significantly increased in the early third instar *fkbp39* mutant suggests that the JH titer is higher in the mutants at that time, since *Kr-h1* expression is thought to be a direct response to JH [13]. This increase in *Kr-h1* likely causes the prolongation of larval life seen in these mutants, since JH acting via Kr-h1 in the prothoracic gland inhibits ecdysteroid biosynthesis [11]. How FKBP39 exerts this effect on JH biosynthesis and/or on *Kr-h1* is not understood, although it has been proposed to act as a transcription factor involved in a multiprotein complex that associates with a JH response element [9]. Further study is needed to elucidate the regulation mechanism of FKBP39 on *Kr-h1* expression.

Both male and female *fkbp39* mutant flies have fertility defects, which suggests that FKBP39 plays an essential role in gamete development, as do other FKBP proteins [25,26,27]. However, the expression of *Kr-h1* was not changed in the *fkbp39* mutant ovaries, meaning that FKBP39 function on fertility might be independent of JH activity. Zhou et al. reported that FKBP39 promoted the degradation of Nprl3, an inhibitor of the target of rapamycin complex 1 (TORC1), and decreased TORC1 activity in the Drosophila ovary [20]. The Nprl3 and TORC1 are essential for Drosophila fertility [28,29]. The question of whether the fertility defects in the fkbp39 mutant are associated with TORC1 requires further study.

Real-time quantitative reverse transcription PCR (qRT-PCR) is an accurate and sensitive method for detecting expression changes at the mRNA level. Because of the multiple steps in the detection process, including RNA isolation, DNase treatment, reverse transcription, and PCR amplification, data normalization using the expression stable reference gene is important for the analysis [30,31]. *rp49* is the most widely used reference mRNA for gene expression studies using the qPCR method in Drosophila [24]. Here, we found that the *rp49* expression was significantly decreased in the *fkbp39* mutant. Our result suggests that *rp49* is not always suitable for normalization in Drosophila studies. How FKBP39 regulates *rp49* expression is an interesting issue for further study. In the larval fat body, we detected the FKBP39 protein in a particular region of the nucleus which is likely the nucleolus. Several other previous studies have shown that FKBP39 localizes primarily in the nucleolus [6,7,8], a place of ribosome assembly, and binds both ribosomal processing proteins and histones [7]. Some FKBPs, like SpFkbp39p and FKBP25, function in ribosome assembly [32,33]. Here, we found that the expression of ribosome protein RPL32 decreased, whereas the expression of RPS20, another ribosome protein component, did not change in the *fkbp39* mutant. Whether FKBP39 has a function in ribosome biogenesis is an interesting issue for further study.

In summary, we have shown the expression pattern and physiological function of FKBP39 in Drosophila. The nucleus-localized FKBP39 inhibits *Kr-h1* expression at the early third larval stage and promotes pupariation, suggesting that FKBP39 plays a role in JH activity. Moreover, FKBP39 is required for fertility. Taken together, FKBP39 plays an essential role in Drosophila development.

## Figures and Tables

**Table 1:** The *fkbp39^1^* is viable.

Cross fkbp39^1^/MKRS	Genotype (Number of Adult Flies) *		Percentage of Expected Ratio
	fkbp39^1^/MKRS	fkbp39^1^	
Repeat 1	245	89	72.6%
Repeat 2	271	127	93.7%
Repeat 3	242	105	86.8%
Repeat 4	145	68	93.8%

* The expected Mendelian ratio of non-Sb to Sb flies was 1:2 since the *MKRS*/*MKRS* is embryonically lethal.

## Data Availability

The data presented in this study are available in article or Supplementary Materials.

## Supplementary Materials

The following supporting information can be downloaded at: https://www.mdpi.com/article/10.3390/insects13040330/s1, Figure S1: Western blot analysis of FKBP39 protein expression in yw (control) and fkbp391 flies. α-Tubulin was used as a loading control. Figure S2: The fat bodies from the second instar larva (64h) of yw and fkbp39 mutant were stained with FKBP39 antibody. The fat bodies were labeled with DAPI (blue) and FKBP39 (red). Bar, 10 m.
