# Supplementary material for: FKBP39 Controls the Larval Stage JH Activity and Development in Drosophila melanogaster
Source: Insects. 2022 Mar 28;13(4):330. doi: 10.3390/insects13040330 (PMC9030728; doi:10.3390/insects13040330)
Supplement: Supplementary file 1 [file insects-13-00330-s001.zip › Figures S1 and S2.pdf]

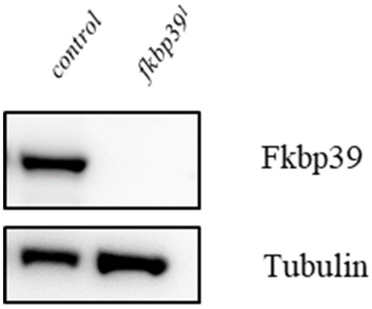

Figure S1. Western blot analysis of FKBP39 protein expression in *yw* (control) and *fkbp39*^{1} flies. α-Tubulin was used as a loading control.

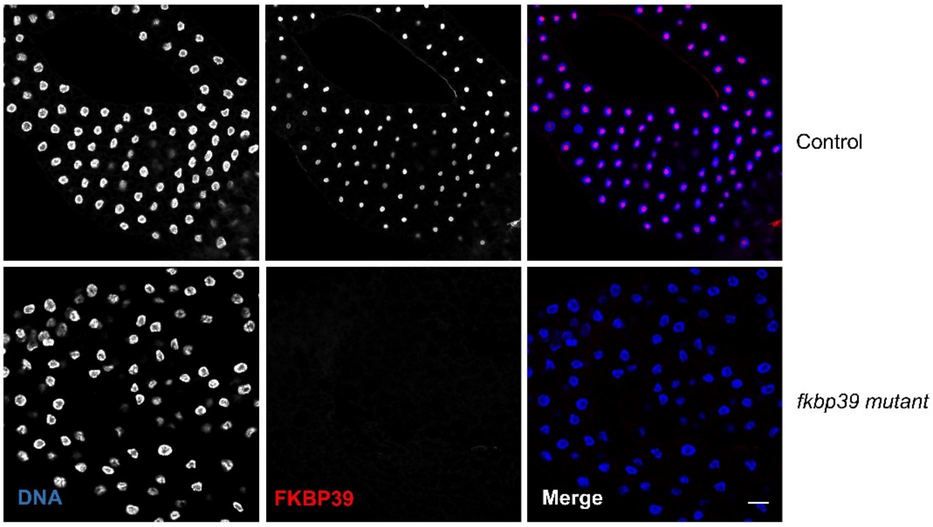

Figure S2. The fat bodies from the second instar larva (64h) of *yw* and *fkbp39* mutant were stained with FKBP39 antibody. The fat bodies were labeled with DAPI (blue) and FKBP39 (red). Bar, 10 µm.
