# Supplementary material for: FKBP39 Controls the Larval Stage JH Activity and Development in Drosophila melanogaster
Source: Insects. 2022 Mar 28;13(4):330. doi: 10.3390/insects13040330 (PMC9030728; doi:10.3390/insects13040330)
Supplement: Supplementary file 1 [file insects-13-00330-s001.zip › The whole blot of Figure 1C and densitometry readingsintensity ratio of each band/Western bolt marker information.pptx]

## Slide 1
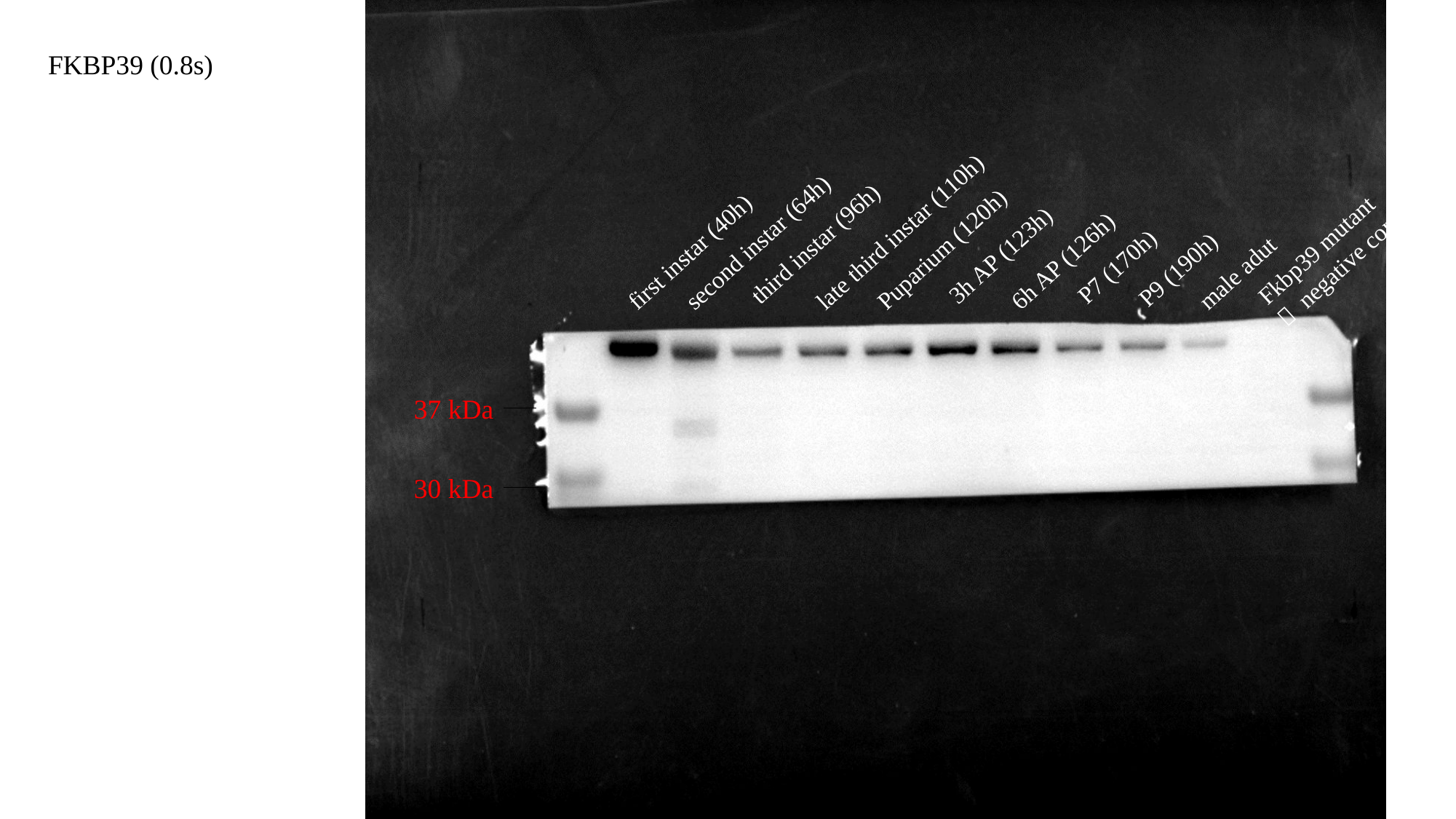

FKBP39 (0.8s)
Fkbp39 mutant （ negative control）
3h AP (123h)
P7 (170h)
P9 (190h)
Puparium (120h)
male adut
third instar (96h)
late third instar (110h)
6h AP (126h)
first instar (40h)
second instar (64h)
37 kDa
30 kDa

## Slide 2
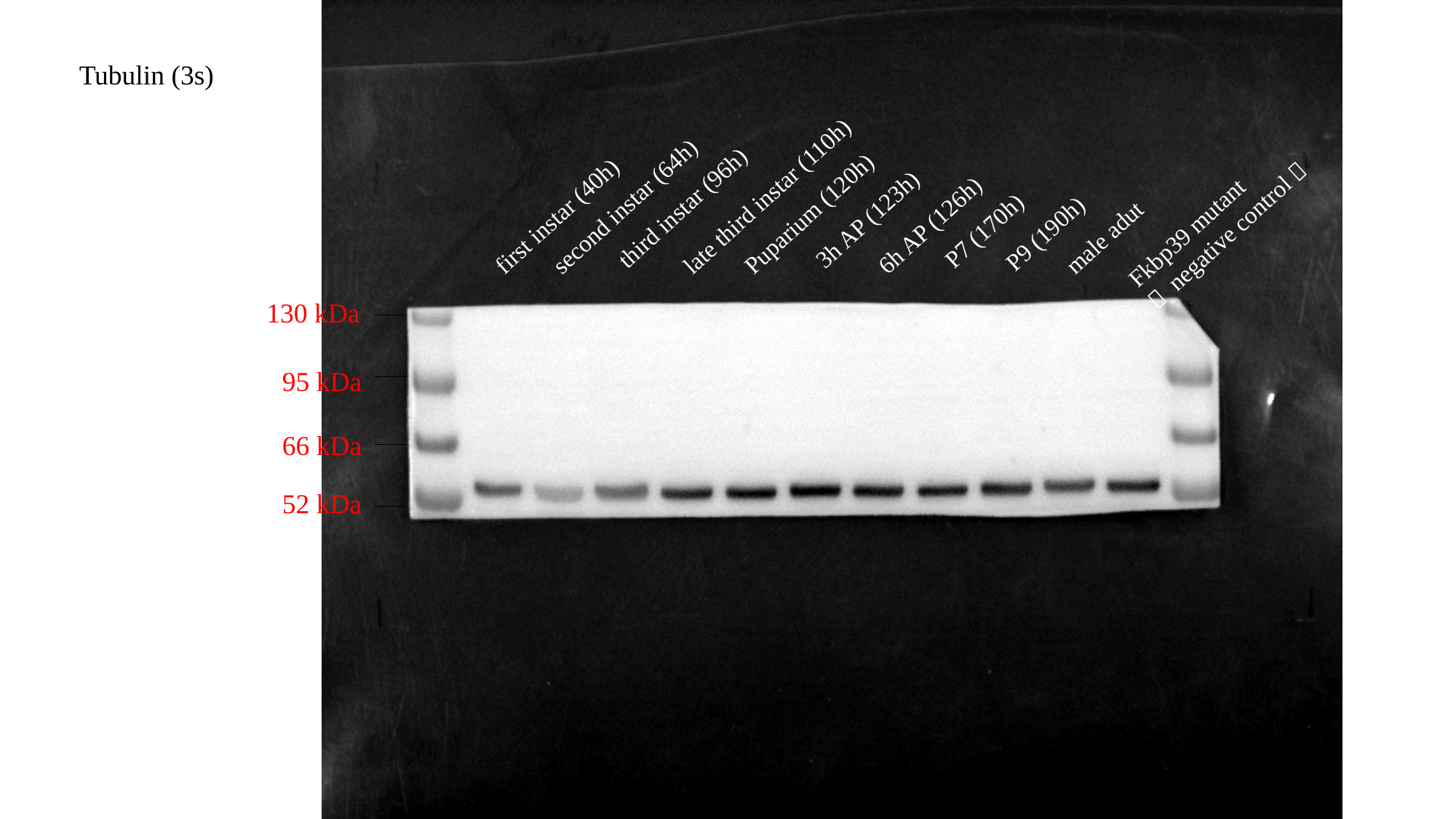

Tubulin (3s)
3h AP (123h)
P7 (170h)
P9 (190h)
Puparium (120h)
male adut
third instar (96h)
late third instar (110h)
6h AP (126h)
first instar (40h)
second instar (64h)
Fkbp39 mutant （ negative control）
130 kDa
95 kDa
66 kDa
52 kDa
