# Supplementary figures and images for: FKBP39 Controls the Larval Stage JH Activity and Development in Drosophila melanogaster
Source: Insects. 2022 Mar 28;13(4):330. doi: 10.3390/insects13040330 (PMC9030728; doi:10.3390/insects13040330)

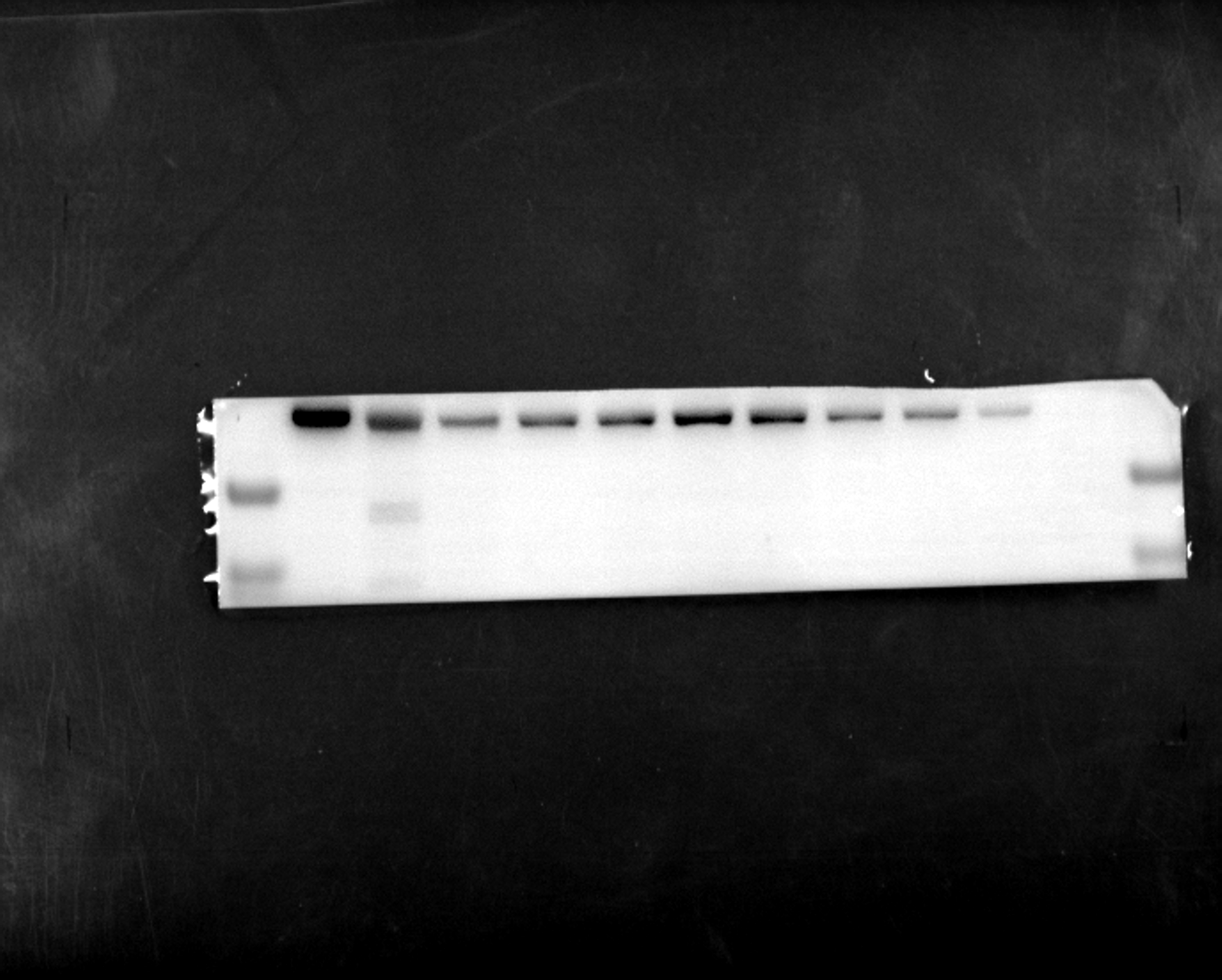

Supplement: Supplementary file 1 [file insects-13-00330-s001.zip › The whole blot of Figure 1C and densitometry readingsintensity ratio of each band/FKBP39 0.8s-merged with marker.bmp]

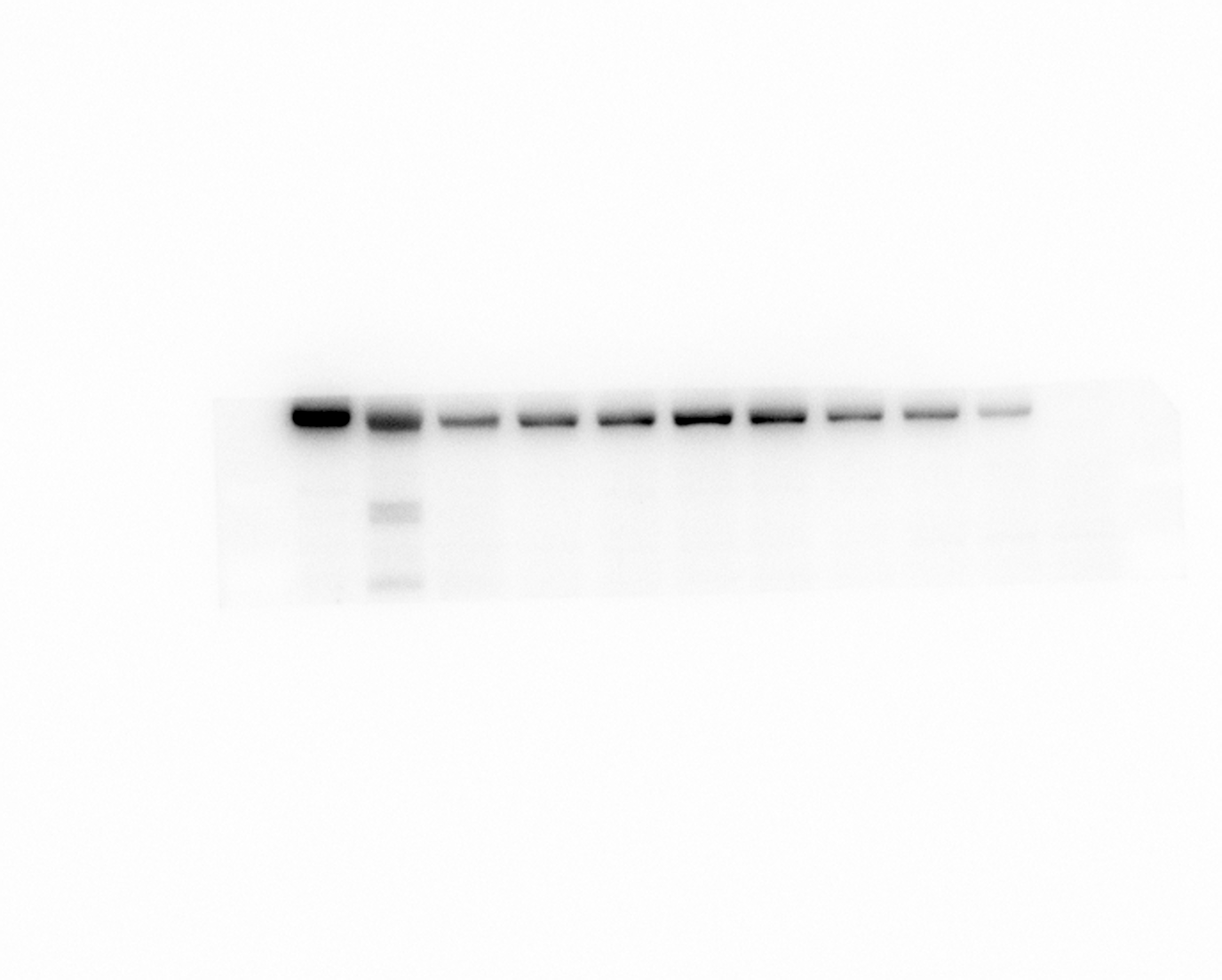

Supplement: Supplementary file 1 [file insects-13-00330-s001.zip › The whole blot of Figure 1C and densitometry readingsintensity ratio of each band/FKBP39 0.8s-used in the paper.bmp]

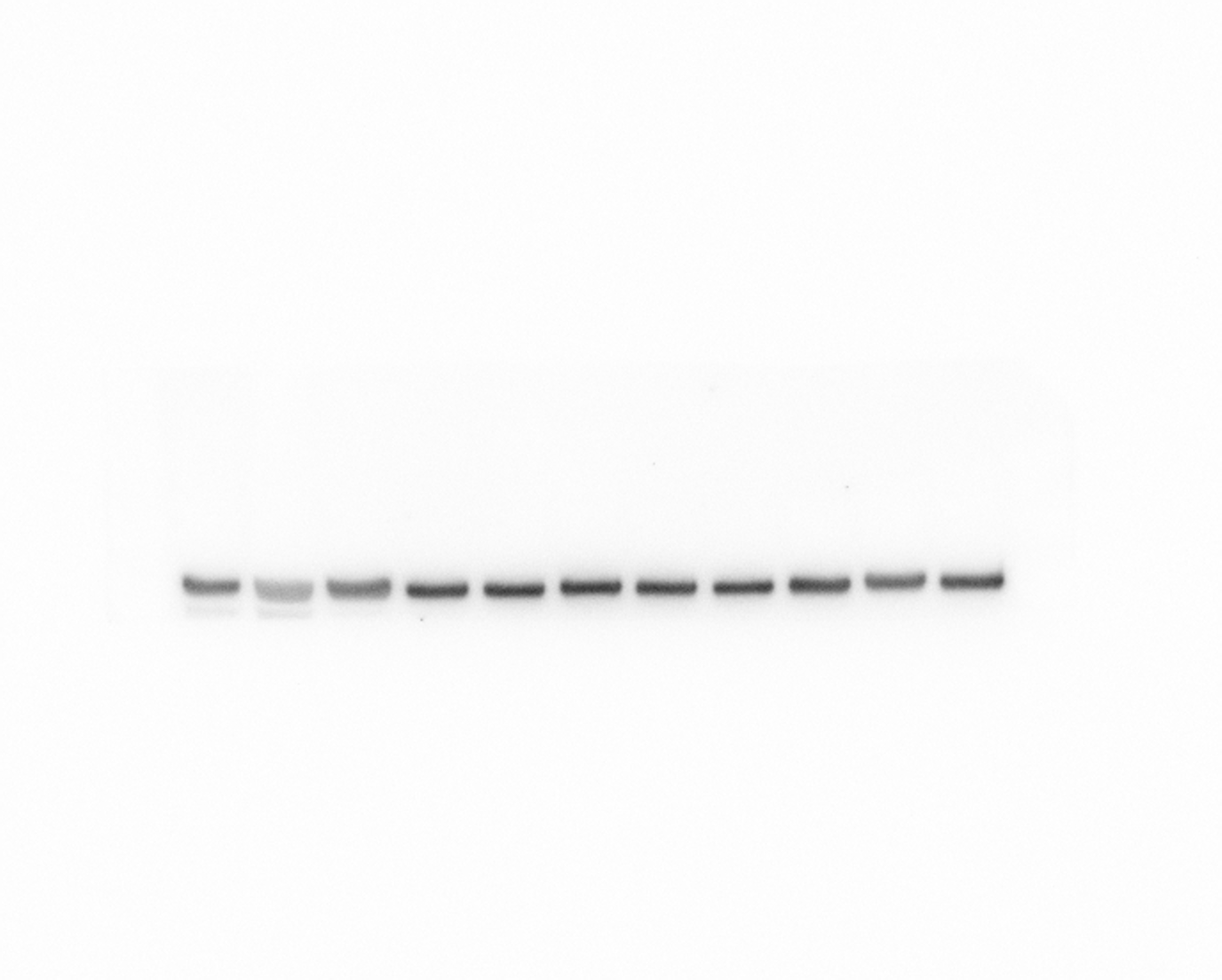

Supplement: Supplementary file 1 [file insects-13-00330-s001.zip › The whole blot of Figure 1C and densitometry readingsintensity ratio of each band/Tubulin 2.5s- used in the paper.bmp]

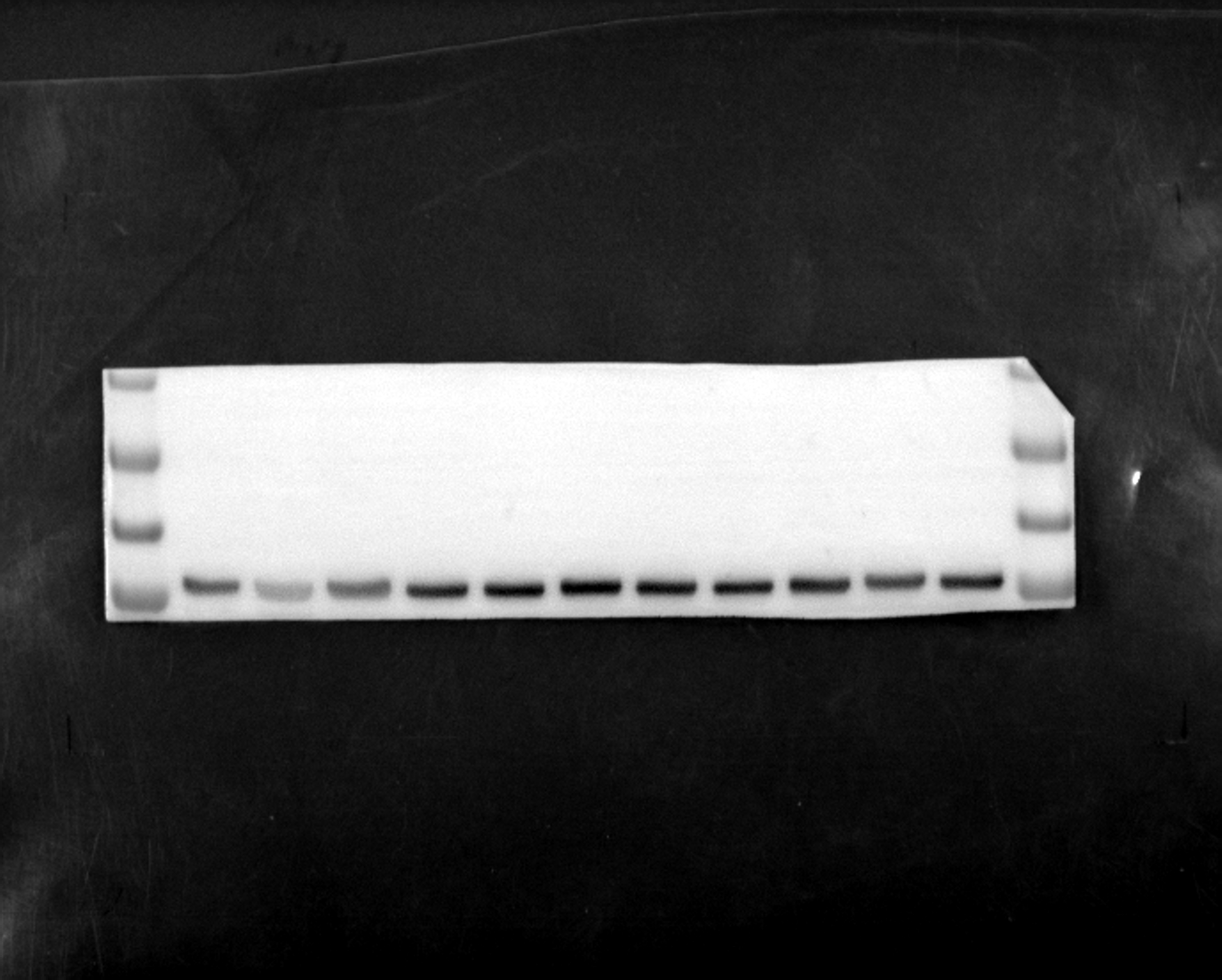

Supplement: Supplementary file 1 [file insects-13-00330-s001.zip › The whole blot of Figure 1C and densitometry readingsintensity ratio of each band/Tubulin 3s-merged with marker.bmp]
